# Patients’ subjective well-being: Determinants and its usage as a metric of healthcare service quality

**DOI:** 10.1177/13591053241246933

**Published:** 2024-04-20

**Authors:** Henry A Lee, Neo Poon, Paul Dolan, Ara Darzi, Ivo Vlaev

**Affiliations:** 1 Imperial College London, UK; 2 University of Bristol, UK; 3 University of Warwick, UK; 4 London School of Economics, UK

**Keywords:** healthcare quality, patient experience, subjective well-being

## Abstract

It is commonly suggested that patients’ subjective well-being (SWB) can be affected by pre-treatment conditions and treatment experiences, and hence SWB can be used to measure and improve healthcare quality. With data collected in a hospital in the UK (*N* = 446), we investigated the determinants of patients’ SWB and evaluated its use in healthcare research. Our findings showed strong relationships between pre-treatment conditions and patients’ SWB: anxiety and depression negatively predicted SWB across all three domains, mobility positively predicted the life satisfaction and happiness domains, while the ability to self care and pain and discomfort also predicted SWB in some domains. In contrast, patients’ satisfaction with the treatment only played minor roles in determining SWB, much less so the characteristics of their nurses. The general lack of associations between treatment experiences and patient’s SWB highlighted the challenges of using SWB to measure healthcare quality and inform policy making.

## Introduction

While it is clear that the quality of healthcare services needs to be measured, there is no consensus on what metrics should be used (Mayer et al., 2009). Traditionally, the effectiveness of treatments has been assessed by clinical outcomes (e.g. mortality, survival or infection rates) or process measures (e.g. staffing levels), which have been criticised for their lack of focus on the lives of the individuals receiving care (Lee et al., 2013; Mickelsson et al., 2022). Recent years have seen an emergence of patient reported outcome measures (PROMs), which are self-reported evaluation of a patient’s condition given a specific treatment (Valderas et al., 2008). However, although PROMs allow patients to provide an assessment on the quality of a particular healthcare service, they seldom capture the overall impacts of a treatment on the patients’ life experience. It has been argued that there is a need for a global metric of experienced utility to appraise the effectiveness of healthcare and assess the interactions between patients and their careers (Chalkidou et al., 2009; Dolan et al., 2009), which led to the recent attention on the subjective well-being (SWB) of patients (Attwood et al., 2020; LaVela and Gallan, 2014; Lepper and McAndrew, 2008).

SWB is an individual’s cognitive and affective judgements of their life (Diener et al., 1999, 2002). In its simplest form, SWB can be measured by simply asking people how well their lives are going. This is closely related to the democratic aspect of preference satisfaction, since it allows individuals to assess the quality of their lives, without someone else doing so (Graham, 2008). In policy research, SWB has been defined over three domains, namely *evaluation*, *experience* and *eudemonia* (Dolan et al., 2011; Dolan and Metcalfe, 2012). Evaluation is the cognitive element of SWB and is the rational judgement of one’s global life satisfaction. Experience is the affective component and refers to an individual’s momentary emotions or feelings, which are often measured by their happiness in the recent past. Finally, eudemonia is defined as high-level psychological needs, including purposes and worthwhileness of life, which influence well-being beyond the utilitarian account of pleasure or pain (Hurka, 1993).

It has been suggested that patients’ SWB is a generalisable metric and allows comparisons between aspects of treatments, conditions and demographic groups which are difficult to perform with domain-specific measures. In other words, SWB is thought to be a commensurable unit to evaluate the effectiveness of clinical practices and provide valuable insights into how their quality can be improved at different stages of patients’ experience (Lee et al., 2013). Finally, it has been noted that PROMs often focus on a micro level to assess new treatments and are not routinely used in most healthcare services, while league tables and resource-based performance markers are used at a macro level, as such patients’ SWB bridges the gap between these metrics.

The leading theoretical account (Lee et al., 2013) suggests that patients’ SWB is affected by three phases of experience: pre-treatment, treatment and post-treatment. Pre-treatment experience is determined by patients’ expectation about the upcoming treatments, anticipation anxiety and, importantly, their pre-existing health conditions. Previous findings showed that lower SWB is associated with poorer reported health (Marmot, 2003), hence the self-perceptions of health states and capabilities in carrying out daily activities should play a crucial role in influencing one’s SWB. Chronic diseases and physical health conditions, especially those which directly threaten the lives of the individuals, can cause rapid declines in SWB (Hudson et al., 2019; Verbrugge et al., 1994). Mental conditions, specifically schizophrenia, depression or anxiety, are also potential risk factors of SWB (Koivumaa-Honkanen et al., 1999; Mukuria and Brazier, 2013; Packer et al., 1997). Treatment experience refers to patient’s satisfaction during their stays at the hospitals, patient-staff relationships and the immediate clinical outcomes. According to the theoretical model, the interpersonal relationships between patients and their carers should have major impacts on their SWB during medical treatments. For instance, the kind acts that are performed by an empathetic nurse in the middle of a hectic shift should dramatically improve a patient’s mood during their stay in a hospital. Similarly, poor encounters with care givers should negatively influence one’s expectation for the remaining duration of their treatment. Despite these seemingly obvious observations, the dynamics between patients’ SWB, their satisfaction with the care which they are receiving, and the characteristics of staff is seldom investigated. Finally, post-treatment experience is related to long term clinical outcomes. Previous findings demonstrated that successful medical treatments can increase SWB (Nilsson et al., 2004) and, in some cases, continue to do so for an entire year (Verbrugge et al., 1994).

The primary goals of this paper are thus threefold and interlinked: the present study investigated the determinants of patients’ SWB in different phases of patient experiences, empirically tested the theoretical model and evaluated the usage of SWB in measuring the quality of healthcare in clinical settings. Although the relationships between pre-treatment conditions and patients’ SWB have been established (Hudson et al., 2019; Marmot, 2003; Mukuria and Brazier, 2013), the links between treatment experience and SWB is less explored and less directly tested, especially the patient-staff aspects. The current body of literature often assumes that the infrastructure and environment of a healthcare system have major impacts on the emotion and experience of the patients, and more importantly, that these effects can be measured by SWB scales which are then used to inform how the limited resources of the ecosystem should be allocated and balanced (Danaher and Gallan, 2016; Jones, 2023; Jones and Drummond, 2021). McColl-Kennedy et al. (2017) suggested that healthcare services are inherently emotional and frequently high-stakes due to the risk of mortality and the invasiveness of clinical procedures, therefore the SWB of patients should be carefully monitored throughout their journeys within the services. Similarly, it has been noted that SWB is one of the tools to monitor and reduce the stress level of patients inside a healthcare system, and can be used to develop the hospitality aspects of the environment which facilitate recovery and provide a sense of control to the patients (Suess and Mody, 2017; Ulrich, 1991), potentially through the inclusion of hotel-style bedding, room service and furniture designs (Mody et al., 2020; Suess and Mody, 2018). Additionally, it has been suggested that the satisfaction of patients can inform policy making and influence healthcare reform by allowing problems and needs to be rapidly identified (Kaushik and Raman, 2015). It is thus pivotal to verify these notions and evaluate the use of patients’ SWB as a metric to measure the quality of healthcare services. A better understanding of patients’ SWB could potentially have important implications on the flow of hospital treatments, the training courses received by staff members and the values of SWB in the realm of healthcare research.

## Methods

### Data collection

#### Data collection approach

The data was collected in a hospital in the United Kingdom at two time points, one in the summer and the other in the winter. This decision was made to increase sample size, as recruiting healthcare staff during their shifts is tremendously difficult and often faces reluctance. All inpatients fulfilling the inclusion criteria, as stated below, and all nurses on duty at the two time points were invited to participate in the study. Data collection took place in the afternoon to prevent interference with morning clinical ward rounds, and each participant was given 3 hours to complete a brief questionnaire.

English-speaking adult inpatients in the surgical, medical and gynaecology wards were eligible in the study. The paediatric population was excluded as they were less suitable for self-reported instruments. To minimise variability in the level of cognition and consciousness, patients admitted to the Intensive Care Unit were excluded. Furthermore, individuals identified by ward nurses as having reduced levels of consciousness, as measured by the Glasgow Coma Scale (Teasdale and Jennett, 1974), were also excluded. Specifically, this refers to patients who did not get the highest responsiveness score possible which is 15. Patients could receive assistance in completing the questionnaire, but they must be able to provide their own responses.

#### Sample size

At each time point, 378 inpatient beds were eligible, after excluding paediatric and critical care patients. 226 patients fulfilled the inclusion criteria of language and responsiveness and agreed to participate at the first time point, 220 at the second time point, totalling 446 patients.

As for nurses, 37 out of 59 nurses on duty agreed to complete the questionnaire at the first time point, 36 out of 54 at the second, totalling 73 nurses. By cross referencing the demographic data at the two time points, we confirmed that no nurse repeatedly participated in the study.

#### Sample characteristics

58.30% of the patients were female and the mean age was 59.07 (SD = 18.25), which means the sample was older than the average British population. Considering that we excluded paediatric patients and that elder individuals are more likely to be admitted to clinical settings, we deem this sample to be appropriate. The majority of patients were not smokers (77.58%), had not undergone surgery during their hospital stay (67.26%) and were not treated for an infection (70.18%).

80.82% of the nurses were female and the mean age was 35.96 (SD = 11.02). The majority of nurses were not smokers (67.12%).

### Measures

#### Subjective well-being

Four items were used to measure the three SWB domains mentioned above. Specifically, the evaluative domain of SWB was measured by asking patients the extent to which (1) they were satisfied with their lives; the experiential domain was measured by one question asking the extent to which (2) they felt happy in the previous day and another question about the extent to which (3) they felt anxious in the previous day; while the eudemonic domain was measured by asking the extent to which (4) they felt the things they did in life were worthwhile. The items were adopted from the Measuring National Well-being Programme in the UK (Office for National Statistics, 2010) and were on 0–10 scales. These items were included in both the patients’ and nurses’ versions of the survey.

#### Health states

Health was mainly measured by EQ5D which is widely used in the British healthcare system (NHS England, 2014). Five core EQ5D items along with the EQ5D Visual Analogue Scale (VAS) were used. The former asked participants to choose the statements which best described five different aspects of their health state, namely (1) mobility, (2) self-care, (3) usual activities, (4) pain and discomfort and (5) anxiety and depression. Each question had three statements from which participants could choose, ranging from the most positive to the least. For instance, the three statements for mobility were ‘I have no problems in walking about’, ‘I have some problems in walking about’ and ‘I am confined to bed’. Furthermore, the VAS provided each participant with a scale which ranged from 0 to 100 points, with 100 points representing the best imaginable health state, and asked them to draw a line from a box labelled ‘your own health state’ to a point on the scale which reflected their current health. Both patients and nurses answered these questions.

#### Patients’ treatment satisfaction

Five questions were used to measure patients’ experience about their treatment. These items were co-developed with the patient experience team within the participating hospital. The five questions asked the extent to which each patient was satisfied with (1) the overall care they had received, (2) the doctors who had treated them, (3) the nurses who had treated them, (4) communication from staff and (5) the extent to which they had been treated with dignity and respect. All questions were on 0–10 scales. Furthermore, we asked about the length of their hospital stay.

Three additional questions related to treatment experience were included. The first two asked a participant about (1) their energy in the previous day and (2) how well they slept in the previous night, both of which were on 0–10 scales. The final item was a binary question asking (3) whether patients were treated for an infection during their hospital stay. Independent of the questionnaire, we also collected information about whether patients had undergone surgery in the hospital.

#### Nurses’ job satisfaction

Three questions were used to measure nurses’ job satisfaction. The first two items were on 0–10 scales and asked the extent to which each participant were (1) satisfied with their job and (2) felt valued by their employer. The final question was a binary choice asking (3) whether they would recommend the hospital to their friends or family members. Additionally, we asked about their years of service.

#### Demographic information

Five items were adopted from the British Household Panel Survey, which asked a participant about their (1) age, (2) gender, (3) marital status, (4) whether they smoked and (5) whether they had children. Both patients and nurses answered these questions.

### Ethical approval

Ethical approval was sought for the study, but the Research Ethics Committee at Imperial College London deemed that none was necessary as no major intervention or change in procedure was being investigated. This study was approved by the patient experience team at the hospital where data collection took place.

## Results

All analyses in this paper were performed with R (R Core Team, 2024) and statistical packages in the R ecosystem.

### Patients’ characteristics

#### Subjective well-being

**Figure 1.**
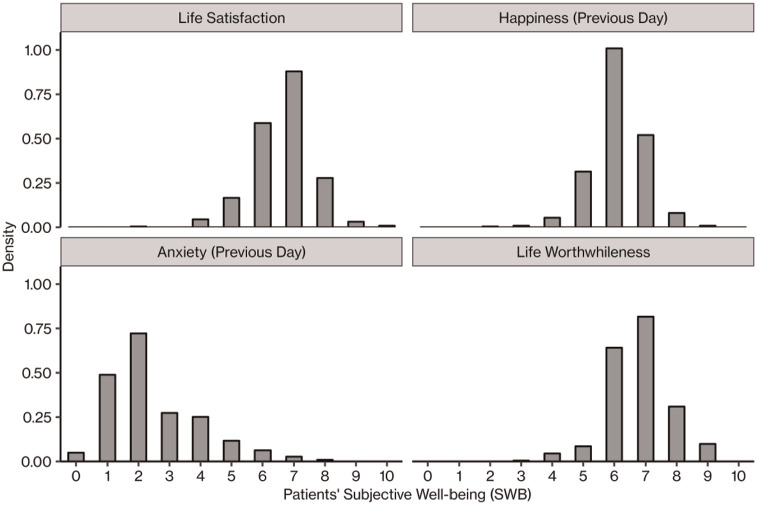
Distributions of patients’ subjective well-being (SWB) on the four domains.

Table 1 shows that patients reported above-average levels of SWB on all four measures. Figure 1 shows their distributions.

**Table 1.** Summary statistics for patients’ SWB.

SWB measures	Mean	Standard deviation
Life satisfaction	6.65	1.00
Happiness (previous day)	6.12	0.89
Anxiety (previous day)	2.49	1.51
Life worthwhileness	6.77	1.02

#### Health

Table 2 shows that the majority of patients were not experiencing severe problems, as reflected by the EQ5D scores. Cronbach’s α of the EQ5D scores is 0.51. Since EQ5D is a heterogeneous inventory and consists of items that address qualitatively different aspects of health states, Cronbach’s α cannot have a very high value (Cronbach, 1951; Konerding, 2013), we therefore deem EQ5D to be appropriate in this study.

**Table 2.** Summary statistics for patients’ health.

EQ5D measures	No problem (%)	Some problems (%)	Severe problems (%)
Mobility	37.44	45.74	16.82
Self-care	34.53	53.14	12.33
Usual activities	32.29	56.50	11.21
Pain and discomfort	41.48	53.14	5.38
Anxiety and depression	66.82	26.91	6.27

For the analyses reported below, the EQ5D measures of mobility, self-care and usual activities were reverse coded in a way such that higher values represented higher levels of health. However, the items of pain and discomfort and anxiety and depression were coded in the opposite way, as such higher values represented health issues of higher severity levels. This is to ease the interpretations of our regression models.

#### Subjective well-being and health

We performed regression analyses on the SWB measures separately, as the items did not sufficiently form a high-level construct (see Supplemental Material A).

The SWB measure of anxiety was not used as a dependent variable in this section, because anxiety and depression was also an item of the EQ5D scales which we used as a predictor. Independent variables in the models included the five EQ5D measures, patients’ treatment satisfaction measures and demographic variables.

##### Life satisfaction

Results of a linear regression showed that three of the EQ5D health measures were related to the life satisfaction domain of patients’ SWB. Specifically, patients who were more mobile (*b* = 0.15, *t* = 2.19, *p* = 0.029, 95% CI [0.02, 0.29]), more capable in taking care of themselves (*b* = 0.18, *t* = 2.18, *p* = 0.030, 95% CI [0.02, 0.33]) and experiencing a lower level of anxiety and depression (*b* = −0.31, *t* = −4.04, *p* < 0.001, 95% CI [−0.46, −0.16]) were more satisfied with their lives. However, whether patients could carry out their usual activities had no effect on their life satisfaction (*b* = 0.03, *t* = 0.38, *p* = 0.707, 95% CI [−0.13, 0.19]), nor did their level of pain and discomfort (*b* = −0.09, *t* = −1.13, *p* = 0.258, 95% CI [−0.24, 0.07]). Additionally, patients who were more satisfied with the overall quality of the care they had received reported higher levels of life satisfaction (*b* = 0.12, *t* = 2.21, *p* = 0.028, 95% CI [0.01, 0.23]), but no other measures of patients’ satisfaction were associated with life satisfaction (satisfaction with doctors: *b* = 0.01, *t* = 0.23, *p* = 0.820, 95% CI [−0.08, 0.10]; satisfaction with nurses: *b* = 0.07, *t* = 1.36, *p* = 0.175, 95% CI [−0.03, 0.16]; satisfaction with communication: *b* = 0.00063, *t* = 0.01, *p* = 0.990, 95% CI [−0.10, 0.10]; dignity and respect: *b* = −0.02, *t* = −0.42, *p* = 0.671, 95% CI [−0.12, 0.08]). Finally, patients who were more energetic in the previous day reported a higher level of life satisfaction (*b* = 0.12, *t* = 3.37, *p* < 0.001, 95% CI [0.05, 0.19]), while smoker also scored lower on this SWB domain (*b* = −0.13, *t* = −2.46, *p* = 0.014, 95% CI [−0.23, −0.03]). The full regression model is in Table S2 of Supplemental Material B.

##### Happiness in the previous day

A linear regression found that two of the EQ5D health measures could predict the happiness aspect patients’ SWB, as such patients who were more mobile (*b* = 0.22, *t* = 3.40, *p* < 0.001, 95% CI [0.09, 0.34]) and felt less anxious or depressed (*b* = −0.14, *t* = −2.07, *p* = 0.040, 95% CI [−0.28, −0.007]) were more happy. The remaining three EQ5D measures had no effect on patients’ happiness (self-care: *b* = 0.15, *t* = 1.96, *p* = 0.051, 95% CI [−0.00075, 0.29]; usual activities: *b* = −0.07, *t* = −0.99, *p* = 0.321, 95% CI [−0.22, 0.07]; pain and discomfort: *b* = 0.04, *t* = 0.53, *p* = 0.594, 95% CI [−0.10, 0.18]). Finally, patients slept better in the previous day also reported a higher level of happiness (*b* = 0.09, *t* = 3.29, *p* = 0.001, 95% CI [0.04, 0.14]), while male patients also reported to be happier (*b* = 0.09, *t* = 2.02, *p* = 0.044, 95% CI [0.0024, 0.17]). Patient experience had no effect on happiness (overall satisfaction with care: *b* = 0.03, *t* = 0.51, *p* = 0.613, 95% CI [−0.07, 0.12]; satisfaction with doctors: *b* = 0.06, *t* = 1.46, *p* = 0.145, 95% CI [−0.02, 0.14]; satisfaction with nurses: *b* = −0.0062, *t* = −0.14, *p* = 0.889, 95% CI [−0.09, 0.08]; satisfaction with communication: *b* = −0.01, *t* = −0.23, *p* = 0.820, 95% CI [−0.10, 0.08]; dignity and respect: *b* = −0.04, *t* = −0.76, *p* = 0.446, 95% CI [−0.13, 0.06]). See Table S3 of Supplemental Material B for the full regression model.

##### Life worthwhileness

With a linear regression, we found that two EQ5D health measures were negatively associated with the eudemonic domain of patients’ SWB. Results showed that patients who were experiencing a lower level of pain and discomfort (*b* = −0.17, *t* = −2.04, *p* = 0.042, 95% CI [−0.34, −0.0066]), as well as those who felt less anxious or depressed (*b* = −0.16, *t* = −2.00, *p* = 0.046, 95% CI [−0.32, −0.0028]), considered life to be more worthwhile. Neither the capability to be mobile (*b* = 0.04, *t* = 0.47, *p* = 0.638, 95% CI [−0.11, 0.18]), to take care of one’s self (*b* = 0.04, *t* = 0.47, *p* = 0.635, 95% CI [−0.13, 0.21]), nor to perform usual activities (*b* = 0.07, *t* = 0.78, *p* = 0.436, 95% CI [−0.10, 0.24]) had any significant effect on patients’ perceived life worthwhileness. Finally, patients’ level of energy was positively related to perceived life worthwhileness (*b* = 0.08, *t* = 1.98, *p* = 0.049, 95% CI [0.00037, 0.15]). None of the metrics related to patient satisfaction influenced the perception of life worthwhileness (overall satisfaction with care: *b* = 0.07, *t* = 1.22, *p* = 0.225, 95% CI [−0.04, 0.19]; satisfaction with doctors: *b* = 0.07, *t* = 1.38, *p* = 0.169, 95% CI [−0.03, 0.16]; satisfaction with nurses: *b* = 0.03, *t* = 0.57, *p* = 0.568, 95% CI [−0.07, 0.13]; satisfaction with communication: *b* = 0.04, *t* = 0.68, *p* = 0.495, 95% CI [−0.07, 0.14]; dignity and respect: *b* = −0.02, *t* = −0.31, *p* = 0.758, 95% CI [−0.12, 0.09]). The full regression model is in Table S4 of Supplemental Material B.

### Nurses’ characteristics

As we were interested in how the characteristics of the nurses could influence patients’ SWB, we computed the average SWB scores, health states and job satisfaction of the nurses in each ward at each time point. All four SWB measures were used for nurses in this section. Health states of nurses were represented by the EQ5D VAS item whose scores were transformed to the scale of 0–10.

Table 3 shows that, when mean-aggregated for each ward at each time point, nurses reported above-average levels of health, SWB and job satisfaction.

**Table 3.** Summary statistics for nurses’ characteristics, aggregated for each ward at each time point.

Measures	Mean	Standard deviation
EQ5D Visual Analogue Scale (VAS)	7.03	0.72
SWB (life satisfaction)	7.02	0.80
SWB (happiness in the previous day)	6.64	0.79
SWB (anxiety in the previous day)	2.22	1.65
SWB (life worthwhileness)	7.55	0.83
Job satisfaction	6.97	0.83

#### Patients’ SWB and nurses’ characteristics

We analysed patients’ SWB measures separately and regressed each of them on the aggregated levels of nurses SWB scores, health states and job satisfaction.

##### Life satisfaction

A linear regression found a significant relationship between the experiential domain of nurses’ SWB and the evaluative domain of their patients’ SWB. Specifically, results showed that patients reported a higher level of life satisfaction when their nurses reported a lower level of anxiety in the previous day (*b* = −0.11, *t* = −2.17, *p* = 0.031, 95% CI [−0.22, −0.01]). However, other domains of nurses’ SWB did not predict their patients’ life satisfaction: neither nurses’ life satisfaction (*b* = 0.02, *t* = 0.17, *p* = 0.866, 95% CI [−0.20, 0.24]), happiness in the previous day (*b* = −0.12, *t* = −0.92, *p* = 0.357, 95% CI [−0.39, 0.14]), nor perceived life worthwhileness (*b* = 0.02, *t* = 0.17, *p* = 0.864, 95% CI [−0.24, 0.28]) had any effect. Nurses’ health states (*b* = 0.03, *t* = 0.31, *p* = 0.759, 95% CI [−0.16, 0.22]) and job satisfaction (*b* = −0.11, *t* = −1.24, *p* = 0.217, 95% CI [−0.30, 0.07]) did not affect their patients’ life satisfaction either.

##### Happiness in the previous day

Results of a linear regression showed that nurses’ health and SWB did not predict the happiness aspect of their patients’ SWB. None of the measures among nurses’ health state scores (*b* = 0.01, *t* = 0.16, *p* = 0.869, 95% CI [−0.15, 0.18]), life satisfaction (*b* = 0.07, *t* = 0.65, *p* = 0.516, 95% CI [−0.13, 0.26]), happiness in the previous day (*b* = 0.05, *t* = 0.38, *p* = 0.704, 95% CI [−0.19, 0.28]), level of anxiety in the previous day (*b* = −0.09, *t* = −1.88, *p* = 0.060, 95% CI [−0.18, 0.0038]), nor perceived life worthwhileness (*b* = −0.14, *t* = −1.22, *p* = 0.225, 95% CI [−0.37, 0.09]) had any relationship with their patients’ happiness in the previous day. However, surprisingly, nurses’ job satisfaction showed a negative relationship with their patients’ happiness (*b* = −0.23, *t* = −2.83, *p* = 0.005, 95% CI [−0.39, −0.07]).

##### Life worthwhileness

Similar to results of patients’ happiness, nurses’ health states were not associated with their patients’ perceived life worthwhileness (*b* = 0.13, *t* = 1.34, *p* = 0.181, 95% CI [−0.06, 0.32]). None of the measures of nurses’ SWB predicted their patients’ life worthwhileness scores (life satisfaction: *b* = 0.05, *t* = 0.41, *p* = 0.680, 95% CI [−0.18, 0.27]; happiness in the previous day: *b* = −0.20, *t* = −1.45, *p* = 0.149, 95% CI [−0.47, 0.07]; level of anxiety in the previous day: *b* = −0.06, *t* = −1.16, *p* = 0.245, 95% CI [−0.17, 0.04]; life worthwhileness: *b* = 0.09, *t* = 0.69, *p* = 0.493, 95% CI [−0.17, 0.35]). Again, there was a negative relationship between nurses’ job satisfaction and their patients’ perceived life worthwhileness (*b* = −0.19, *t* = −2.00, *p* = 0.046, 95% CI [−0.37, −0.0031]).

## Discussion

Subjective well-being (SWB) involves three domains, which are evaluative (life satisfaction), experiential (happiness in the recent past) and eudemonic (life worthwhileness) respectively. With a unique data set collected in a clinical setting, this work examines the determinants of patients’ SWB across its domains during their hospital stays. Specifically, linking to the theoretical account of patients’ SWB proposed by Lee et al. (2013), we investigated determinants in the pre-treatment and treatment phases of patient experiences, and therefore also evaluate the use of patients’ SWB as a tool to understand the quality of healthcare and inform service improvement.

Our results demonstrated a strong link between pre-treatment conditions and patients’ SWB, with the roles of mental health states being especially noticeable: the feeling of anxiety and depression was negatively associated with all three SWB domains, which is consistent with previous studies (Graham et al., 2009, 2011). Physical health states were also central to well-being, as the capabilities to physically move and care for one self positively predicted life satisfaction. In the case of mobility, it also had a positive relationship with the happiness of patients. Finally, the subjective level of pain and discomfort was negatively correlated with the perception of life worthwhileness, which is also in line with previous work (Dolan and Metcalfe, 2012).

The major role of anxiety and depression in patients’ SWB has clear implications for policy makers and those in clinical roles caring for patients. While a stay at an acute hospital might not provide direct mental health service for inpatients, it is important to understand that patients might be suffering from more than one problem, some of which are less noticeable. For those patients who self-report anxiety and depression, it is prominent to follow up on their states of mental health as it has such a strong impact on SWB. Initiatives, such as local or national programmes, that could help screen for and pro-actively treat mental health conditions within hospitals should be considered as a priority.

Our findings also highlight the importance of improving mobility in clinical populations in order to help address patients’ SWB. A reduction in mobility due to illness is common and not always apparent, while the effect can be long term. Despite individuals often adapt to their difficulties in moving by adjusting their lifestyles, such as catching the bus into town rather than walking, our results demonstrated that it can have major impacts on life satisfaction and perceived life worthwhileness. All acute hospitals have dedicated teams led by physiotherapists that tailor exercises and care plans to improve mobility alongside treatment of any medical problem, which should be utilised.

Linking back to the theoretical model, our results found only limited support for the roles of treatment experience in determining patients’ SWB, especially the interpersonal relationship between patients and their carers. Although our survey included five items directly related to patients’ satisfaction during their stay at the hospital, we found only one relationship: a positive correlation between their overall satisfaction with the care they received and their perception of life satisfaction. However, we found that the subjective energy level of patients and how well they slept in the previous night were related to their SWB. Energy level was positively correlated with patients’ life satisfaction and perceived life worthwhileness, which are results worthy of further exploration. Sleep quality is arguably a measure of patient experience and had a positive relationship with patients’ happiness, which is consistent with previous findings showing that hotel-style bedding and room services can improve patients’ SWB (Mody et al., 2020; Suess and Mody, 2018). Sleeping time is a scarce resource in hospitals (Southwell and Wistow, 1995a, 1995b) and there has been great interest in improving the clinical environments in this aspect. A number of initiatives have been suggested, such as consolidating the number of nursing rounds that are made overnight or having an effective alarm silencing system for non-critical devices, which have minimal cost implications. Eye mask and ear plugs can also greatly improve the quality and length of sleep during hospital stays (Yazdannik et al., 2014). Finally, structural considerations such as the size of hospital wards and the number of patients sharing a room can also have influence on the sleep quality of patients. There might be little incentives for large scale hospital redesign for the purposes of sleep improvement, but our findings certainly support initiatives that seek to improve sleep quality when new units or hospitals are planned.

Our analyses also investigated what characteristics of nurses could predict the well-being of their patients, and we found a negative relationship between nurses’ anxiety level and patients’ life satisfaction. This is a new finding, which naturally has implications that will support the mental health of nurses in clinical work. The reasons behind this relationship could be in part explained by the fact that nurses with a lower level of anxiety are more able to perform their job, which is often strenuous and stressful. Nurses with better well-being may also be more pro-social and likely to engage with patients, which may result in improved SWB for patients. This result is also interesting from a policy perspective, as improvements in nurses’ mental health would appear to help benefit patients in terms of their SWB, which highlighted the values of schemes such as access to occupational mental health services or behaviour change programmes that incentivise exercise or meditation (Dolan et al., 2012; Vlaev et al., 2016).

Taken together, however, our results showed a general lack of relationships between patients’ SWB in a hospital and their satisfaction with their doctors, nurses or communication, nor with the characteristics of their nurses on most domains. This poses a challenge to the theoretical model (Lee et al., 2013), which suggests that treatment experience is an important element in determining patients’ SWB and hence SWB can be practically used to evaluate healthcare quality. While the actions performed by carers in clinical settings are undoubtedly crucial in patients’ experience, it is possible that their impacts on patients’ SWB are overshadowed by pre-existing physical and mental health conditions, as well as the quality of hospital facilities (as reflected by, e.g. sleep quality). Since our results could not consistently demonstrate that different levels of service satisfaction or patient-staff relationships can impact patients’ SWB, they therefore can only provide limited evidence to support patients’ SWB as a reliable metric to capture the perceived quality of healthcare during patients’ stay in a hospital. These findings in this paper are worth replicating, either with longitudinal methods to investigate the extent to which patients’ SWB changes after treatment, or with different measures of patients’ SWB.

Finally, a surprising result in the paper is the negative relationships between nurses’ job satisfaction and their patients’ SWB. While counterintuitive, an explanation is that job satisfaction of healthcare staffs captures their perceived level of job efficiency, which can have a negative impact on patients’ SWB, especially their happiness during their stay at a hospital. This clearly requires further investigation, although these results generally emphasised the need of training programmes which focus on the lives of the patients receiving care.

## Supplemental Material

sj-pdf-1-hpq-10.1177_13591053241246933 – Supplemental material for Patients’ subjective well-being: Determinants and its usage as a metric of healthcare service quality Supplemental material, sj-pdf-1-hpq-10.1177_13591053241246933 for Patients’ subjective well-being: Determinants and its usage as a metric of healthcare service quality by Henry A Lee, Neo Poon, Paul Dolan, Ara Darzi and Ivo Vlaev in Journal of Health Psychology
