# Supplementary material for: Patients’ subjective well-being: Determinants and its usage as a metric of healthcare service quality
Source: J Health Psychol. 2024 Apr 20;30(3):486–97. doi: 10.1177/13591053241246933 (PMC11894880; doi:10.1177/13591053241246933)
Supplement: sj-pdf-1-hpq-10.1177_13591053241246933 – Supplemental material for Patients’ subjective well-being: Determinants and its usage as a metric of healthcare service quality [file sj-pdf-1-hpq-10.1177_13591053241246933.pdf]

Supplementary Materials

Supplementary Materials

Supplementary Material A

Correlations between Patients' SWB Measures

As shown below in Table S1, life satisfaction was positively correlated with happiness and life worthwhileness, and negatively correlated with anxiety. The correlations between the other three SWB variables were weaker.

Table S1

Correlations between the four metrics of patients' SWB.

	Life Satisfaction	Happiness	Anxiety	Life Worthwhileness
Life Satisfaction	-	.37	-.24	.30
Happiness (Previous Day)	-	-	-.15	.07
Anxiety (Previous Day)	-	-	-	-.13
Life Worthwhileness	-	-	-	-

Confirmatory Factor Analysis on Patients' SWB and Health

We tested whether the SWB and EQ5D items converged into high-level constructs with confirmatory factor analysis (CFA). When loading all four SWB items onto a factor and all five EQ5D items onto another factor, the CFA model showed poor fit (CFI = 0.73, RMSEA = 0.11) given the sample size and the number of parameters. Additionally, the standardised loadings were weak for all SWB items (< 0.5) and two EQ5D items (-0.05 for Pain and Discomfort, -0.25 for Anxiety and Depression). Finally, all composite reliability measures were low (< 0.7) for both constructs, so did average variance extracted (AVE; < 0.5).

Supplementary Material B

Regression Models of the Effect of Patients' Health on SWB

Tables S2, S3, and S4 show the full regression models with which we tested the effects of patients' health states, satisfaction with care, and other characteristics on three domains of their SWB.

Table S2

The effects of patients' health states and other characteristics on their life satisfaction.

	Dependent variable:	
	Patients' Life Satisfaction	
	Model 1	Model 2
	(1)	(2)
EQ5D (Mobility)	0.15**	0.15**
	(0.07)	(0.07)
EQ5D (Self-Care)	0.18**	0.18**
	(0.08)	(0.08)
EQ5D (Usual Activities)	0.08	0.03
	(0.08)	(0.08)
EQ5D (Pain and Discomfort)	−0.12	−0.09
	(0.08)	(0.08)
EQ5D (Anxiety and Depression)	−0.36***	−0.31***
	(0.07)	(0.08)
Overall Satisfaction with Care		0.12**
		(0.05)
Satisfaction with Doctors		0.01
		(0.05)
Satisfaction with Nurses		0.07
		(0.05)
Satisfaction with Communication		0.001
		(0.05)
Dignity and Respect		−0.02
		(0.05)
Energy in Previous Day		0.12***
		(0.04)
Sleep Quality in Previous Night		0.03
		(0.03)
Undergone Surgery (Yes vs No)		−0.02

		(0.05)
Treated for Infection (Yes vs No)		0.04
		(0.05)
Gender (Male vs Female)		0.06
		(0.05)
Age		0.002
		(0.002)
Smoker (Yes vs No)		−0.13**
		(0.05)
Martial Status (Married vs Others)		0.05
		(0.05)
Parental Status (Yes vs No)		0.06
		(0.05)
Intercept	6.42***	4.23***
	(0.27)	(0.52)
Observations	446	445
R^2	0.12	0.20
Adjusted R^2	0.11	0.17
Residual Std. Error	0.94 (df = 440)	0.92 (df = 425)
F Statistic	11.97*** (df = 5; 440)	5.63*** (df = 19; 425)
Note:		*p<0.1; **p<0.05; ***p<0.01

Table S3

The effects of patients' health states and other characteristics on their happiness.

	Dependent variable:	
	Patients' Happiness	
	Model 1	Model 2
	(1)	(2)
EQ5D (Mobility)	0.20***	0.22***
	(0.06)	(0.06)
EQ5D (Self-Care)	0.15**	0.15*
	(0.07)	(0.07)

EQ5D (Usual Activities)	−0.03<br>(0.07)	−0.07<br>(0.07)
EQ5D (Pain and Discomfort)	0.04<br>(0.07)	0.04<br>(0.07)
EQ5D (Anxiety and Depression)	−0.20***<br>(0.07)	−0.14**<br>(0.07)
Overall Satisfaction with Care		0.03<br>(0.05)
Satisfaction with Doctors		0.06<br>(0.04)
Satisfaction with Nurses		−0.01<br>(0.04)
Satisfaction with Communication		−0.01<br>(0.05)
Dignity and Respect		−0.04<br>(0.05)
Energy in Previous Day		0.03<br>(0.03)
Sleep Quality in Previous Night		0.09***<br>(0.03)
Undergone Surgery (Yes vs No)		0.08*<br>(0.05)
Treated for Infection (Yes vs No)		−0.03<br>(0.05)
Gender (Male vs Female)		0.09**<br>(0.04)
Age		−0.002<br>(0.002)
Smoker (Yes vs No)		−0.07<br>(0.05)

Martial Status (Married vs Others)		0.04
		(0.04)
Parental Status (Yes vs No)		−0.01
		(0.05)
Intercept	5.61***	4.86***
	(0.24)	(0.48)
Observations	446	445
R^2	0.07	0.13
Adjusted R^2	0.06	0.10
Residual Std. Error	0.86 (df = 440)	0.85 (df = 425)
F Statistic	6.94*** (df = 5; 440)	3.45*** (df = 19; 425)
Note:		*p<0.1; **p<0.05; ***p<0.01

Table S4

The effects of patients' health states and other characteristics on their perceived life worthwhileness.

	Dependent variable:	
	Patients' Life Worthwhileness	
	Model 1	Model 2
	(1)	(2)
EQ5D (Mobility)	0.02	0.04
	(0.07)	(0.07)
EQ5D (Self-Care)	0.06	0.04
	(0.09)	(0.09)
EQ5D (Usual Activities)	0.13	0.07
	(0.08)	(0.09)
EQ5D (Pain and Discomfort)	−0.18**	−0.17**
	(0.08)	(0.08)
EQ5D (Anxiety and Depression)	−0.18**	−0.16**
	(0.08)	(0.08)
Overall Satisfaction with Care		0.07
		(0.06)

Satisfaction with Doctors		0.07
		(0.05)
Satisfaction with Nurses		0.03
		(0.05)
Satisfaction with Communication		0.04
		(0.05)
Dignity and Respect		−0.02
		(0.05)
Energy in Previous Day		0.08**
		(0.04)
Sleep Quality in Previous Night		0.03
		(0.03)
Undergone Surgery (Yes vs No)		0.08
		(0.05)
Treated for Infection (Yes vs No)		0.08
		(0.05)
Gender (Male vs Female)		0.03
		(0.05)
Age		0.001
		(0.003)
Smoker (Yes vs No)		−0.07
		(0.06)
Martial Status (Married vs Others)		−0.04
		(0.05)
Parental Status (Yes vs No)		−0.01
		(0.05)
Intercept	6.87***	5.10***
	(0.28)	(0.56)
Observations	446	445

R^2	0.04	0.10
Adjusted R^2	0.03	0.06
Residual Std. Error	1.00 (df = 440)	0.99 (df = 425)
F Statistic	3.78*** (df = 5; 440)	2.50*** (df = 19; 425)
Note:		*p<0.1; **p<0.05; ***p<0.01
